# Bacterial abundance and diversity in pond water supplied with different feeds

**DOI:** 10.1038/srep35232

**Published:** 2016-10-19

**Authors:** Ya Qin, Jie Hou, Ming Deng, Quansheng Liu, Chongwei Wu, Yingjie Ji, Xugang He

**Affiliations:** 1 Fisheries College, Huazhong Agricultural University, Wuhan 430070, China; 2 Freshwater Aquaculture Collaborative Innovation Center of Hubei Province, Wuhan 430070, China; 3 Hubei Provincial Engineering Laboratory for Pond Aquaculture, Wuhan 430070, China

## Abstract

The abundance and diversity of bacteria in two types of ponds were investigated by quantitative PCR and Illumina MiSeq sequencing. The results revealed that the abundance of bacterial 16S rRNA genes in D ponds (with grass carp fed sudan grass) was significantly lower than that in E ponds (with grass carp fed commercial feed). The microbial communities were dominated by *Proteobacteria*, *Cyanobacteria*, *Bacteroidetes*, and *Actinobacteria* in both E and D ponds, while the abundance of some genera was significantly different between the two types of ponds. Specifically, some potential pathogens such as *Acinetobacter* and *Aeromonas* were found to be significantly decreased, while some probiotics such as *Comamonadaceae unclassified* and *Bacillales* unclassified were significantly increased in D ponds. In addition, water quality of D ponds was better than that of E ponds. Temperature, dissolved oxygen and nutrients had significant influence on bacterial communities. The differences in bacterial community compositions between the two types of ponds could be partially explained by the different water conditions.

Aquaculture is currently among the fastest-growing sector of food production in the world^1^. Global aquaculture production has been growing at an average rate of 6.2% per year during 2000–2012. It was reported that aquaculture production might account for 62% of the total amount of fish consumed by human beings in 2030^2^. Although the high-density, high-yield aquaculture is rapidly growing in recent years, the development of intensive aquaculture was restricted due to increasing pollution of aquatic environments. One major problem in intensive aquaculture is the accumulation of toxic ammonia and nitrite in water column, which not only reduce water quality but also cause disease or even death of the farmed animals^3^.

In recent years, numerous studies have been reported that the growth of heterotrophic microorganisms will be stimulated and nitrogen is utilized to generate microbial proteins if organic carbon sources are added to the aquaculture systems^4^. With additional organic carbon, an accumulation of microorganism, protozoa and algae will develop in the aquaculture systems^5^. The resulting intensive microbial community can improve water quality through removal of toxic nitrogen species such as ammonia and nitrite, whilst the microbial protein produced can be used as feed^6^. Given the great advantages of adding carbon sources into aquaculture systems, numerous studies have investigated effects of carbon sources addition on water quality control^7^,^8^, protein nutritional contribution^7^,^9^, and growth-promoting for the feed animals^10^. However, there is a lack of information on the effect of adding carbon sources on the overall diversity and composition of bacterial communities in aquaculture ponds. An in-depth analysis of microbial communities will be useful for controlling the bacterial community composition for both optimal water quality and optimal feeding of cultured animal, ultimately for animal’s health.

Traditionally, conventional microbiological techniques, such as cultivation methods, 16S rRNA clone libraries, polymerase chain reaction-denaturing gradient gel electrophoresis (PCR-DGGE) and terminal restriction fragment length polymorphism (T-RFLP) have been used for investigation of bacterial communities in aquaculture^11^,^12^,^13^,^14^,^15^. However, these techniques are often laborious, time-consuming and poorly present the diversity of rare bacterial groups. Recently, high-throughput sequencing has been brought in as a new generation sequencing technology, which enables more detailed profiling of microbial populations with high throughput and low cost^16^,^17^,^18^. Several previous studies have been reported the microbial diversity and abundance in different ecosystems by high-throughput sequencing, such as in marine water^19^, natural wetland^20^, soil^21^, drinking water^22^, activated sludge^23^ and hot spring^24^. Therefore, these recent advances in DNA sequencing technologies allow us to develop a broader understanding of complex microbial communities affected by adding carbon sources into aquaculture system.

In this study, we hypothesized that adding carbon sources into aquaculture system will not only improve water quality but also change microbial communities in water column. To validate this hypothesis, we compared three typical ponds using commercial feed (control) with two ponds using sudan grass as a carbon source in the Gong’an county of Jingzhou, Hubei Province, China. Water samples were collected every month from June to October 2014 to explore the microbial communities using MiSeq Illumina sequencing technique. The findings of this study will help to understand the impact of the additional carbon sources on microbial communities in aquaculture ponds, and evaluated the impact of adding carbon sources on culture environment.

## Results

### Environmental description

**Figure 1:**
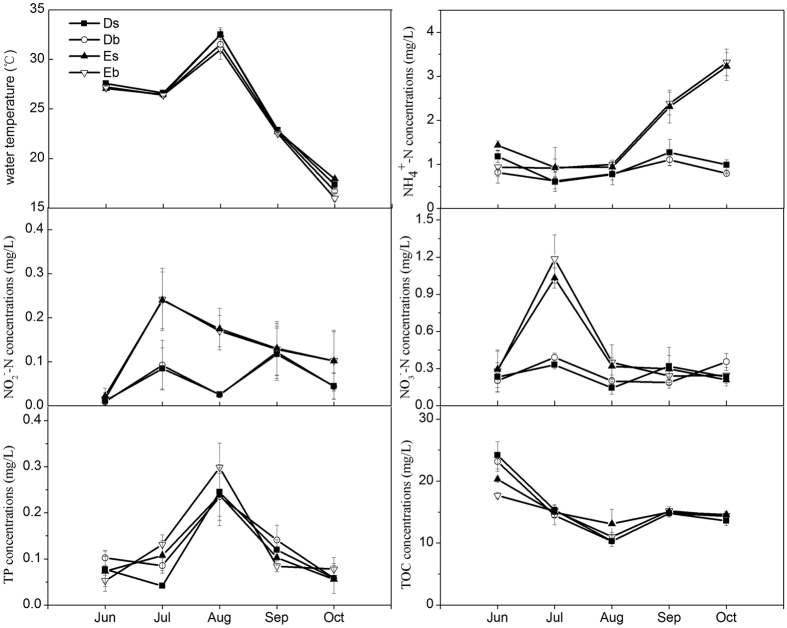
Means ± standard deviations of the of water temperature, NH_4_^+^-N, NO_2_^−^-N, NO_3_^−^-N, TP, and TOC. Concentrations in water layers of two types of ponds over five months (The capital letters D and E represent the ponds in which grass carp were fed with sudan grass and commercial feed, respectively. The lower case s and b represent the surface and bottom water layer, respectively). Error bars represent standard deviations.

The characteristics of the surface and bottom water samples from two different feeding types of ponds within five months are summarized in Table 1. As observed, there were no significant differences in the pH values and DO concentrations no matter between surface and bottom or between two types of ponds (*p* > 0.05). The NH_4_^+^-N and NO_2_^−^-N concentration were also found no significant differences between the surface and bottom from the two type of ponds (*p* > 0.05). However, both NH_4_^+^-N and NO_2_^−^-N concentrations were found significantly lower in the ponds D than that in the ponds E (*p* < 0.05). Variations of water temperature, and the NH_4_^+^-N, NO_2_^−^-N, NO_3_^−^-N, TP, and TOC concentrations of the two types of ponds through five months are shown in Fig. 1. Temperature of the pond water ranged from16.9 ± 0.25 to 32.4 ± 0.06 °C, with the highest record in August. Concentrations of ammonia and nitrite exhibited the same variations in the grass carp fed with sudan grass (D) and those fed with commercial feeds (E). Ammonia and nitrite concentrations were higher in Ponds E than those in Ponds D.

### Abundance of bacterial 16S rRNA genes

**Figure 2:**
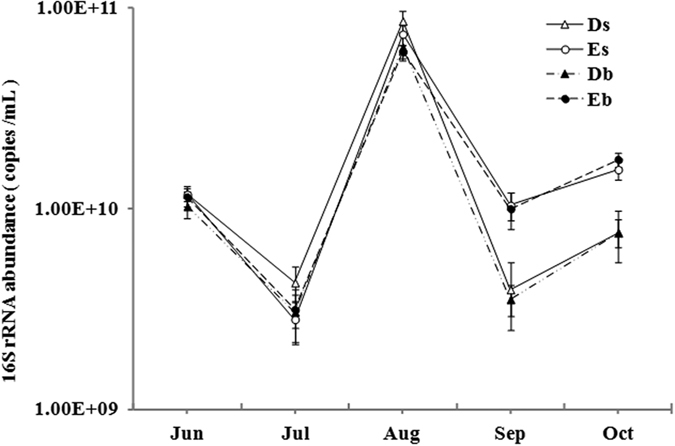
Abundance of bacterial 16S rRNA genes in the surface and bottom water (The capital letters D and E represent the ponds in which grass carp were fed with sudan grass and commercial feed, respectively. The lower case s and b represent the surface and bottom water layer, respectively). Error bars represent standard deviations.

The abundance of bacterial 16S rRNA genes is shown in Fig. 2 as determined by qPCR from two types of ponds. The abundance of bacterial 16S rRNA genes in all samples had the same changing trend: it showed a decrease first, followed by an increase, a decrease and then an increase again (Fig. 2). The bacterial abundance ranged from3.53 ± 0.62 × 10^9^ to 8.63 ± 1.01 × 10^10^ copies/mL and from2.81 ± 0.64 × 10^9^ to 7.42 ± 0.87 × 10^10^ copies/mL for D ponds and E ponds, respectively.

In D ponds, the abundance of bacterial 16S rRNA genes was significantly higher in the surface water layer than in the bottom layer in August (*p* < 0.05), while no significant differences were observed between them in other months (*p* > 0.05). Similar results were obtained for E ponds.

The grass carp were fed with sudan grass in July and August in D ponds, and there were no significant differences in the abundance of bacterial 16S rRNA genes between D ponds and E ponds in these two months (*p* > 0.05). However, in September when the feed was changed to commercial feed again, the abundance of bacterial 16S rRNA genes in D ponds was significantly lower than that of E ponds (*p* < 0.05), and no differences were observed between the two types of ponds in October with further supplement of commercial feed (*p* > 0.05).

### Illumina MiSeq sequencing results

**Figure 3:**
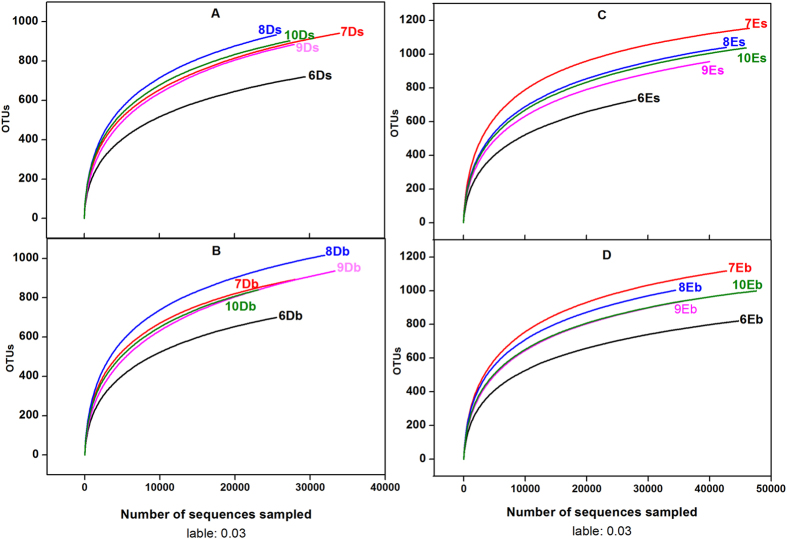
Rarefaction curves for a dissimilarity of 3% from twenty samples. (**A**) The surface water samples of the ponds in which grass carp were fed with sudan grass. (**B**) The bottom water samples of the ponds in which grass carp were fed with sudan grass. (**C**) The surface water samples of the ponds in which grass carp were fed with commercial feed. (**D**) The bottom water samples of the ponds in which grass carp were fed with commercial feed.

After the removal of unqualified reads, the Illumina MiSeq sequencing analysis of fifty water samples yielded 745,337 sequences, with an average length of about 396 bp in the V4–V5 hypervariable regions of the 16S rRNA gene. As summarized in Table 2, the number of operational taxonomy units (OTUs) in the E ponds was larger than that in D ponds no matter in the surface or bottom. By contrast, the Shannon indices showed higher bacterial diversity in D ponds than those in E ponds both on the surface and bottom. It was also noted that bacterial diversity in D ponds was greater than that in E ponds at the bottom layer in August. The rarefaction curves of the twenty samples at the 3% distance cutoff level revealed that the bacterial phylotype richness of sample 7Es was considerably higher than that of other samples (Fig. 3).

### Bacterial community composition

**Figure 4:**
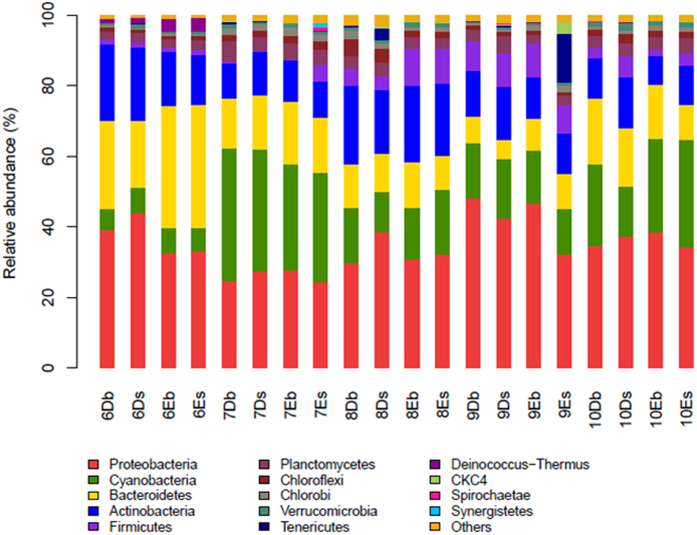
Relative abundance of bacterial community composition in twenty samples at the phylum level over five months (The number before the sample name represents the sampling month). The capital letters D and E represent the ponds in which grass carp were fed with sudan grass and commercial feed, respectively. The lower case s and bin the sample name represent the surface and bottom water layer, respectively). Taxa represented occurred at >1% abundance in at least one sample.

RDP Classifier was used to assign the effective sequence tags into different phylogenetic bacterial taxa. Figure 4 shows the relative abundance of bacterial community at the level of phylum. *Proteobacteria*, *Cyanobacteria*, *Bacteroidetes* and *Actinobacteria* were four phyla abundant in all the samples from the two types of ponds. Except for July, *Proteobacteria* were the most abundant phylum in all experimental months, accounting for 34.95% of the total effective bacterial sequences, followed by *Cyanobacteria* (averaging at 18.8%), *Bacteroidetes* (averaging at 15.8%) and *Actinobacteria* (averaging at 14.7%). Other major phyla (average abundance  > 1%) of the bacterial communities of the twenty samples included *Firmicutes* (4.6%), *Planctomycetes* (3.5%), *Chloroflexi* (1.9%) and *Chlorobi* (1.2%). *Proteobacteria*, *Bacteroidetes*, and *Actinobacteria* had the same varying tendency during the five months at the four sampling sites. However, in the surface water layer of D ponds, *Cyanobacteria* were first increased and then decreased during August to October, while in other three sampling sites it had the opposite varying tendency.

To further understand the differences in dominant phyla between D and E ponds, the mean relative abundance of the four dominant OTUs at each sampling site in the five months was analyzed (Supplementary Fig. S1). *Proteobacteria* were found to be the most dominant phylum in all samples (except for the samples in July) followed by *Actinobacteria*, *Bacteroidetes* and *Cyanobacteria*. As observed, the relative abundance of *Proteobacteria* and *Actinobacteria* in D ponds were both higher than those in E ponds. However, the relative abundance of *Bacteroidetes* in D ponds was lower than that in E ponds. The relative abundance of *Cyanobacteria* fluctuated in D and E ponds, and showed no particular trends. For the rest, *Firmicutes* and *Chlorobi* were more abundant in E ponds than that in D ponds (4.0% and 1.1%, respectively), whereas *Chloroflexi* and *Planctomycetes* were less abundant.

**Figure 5:**
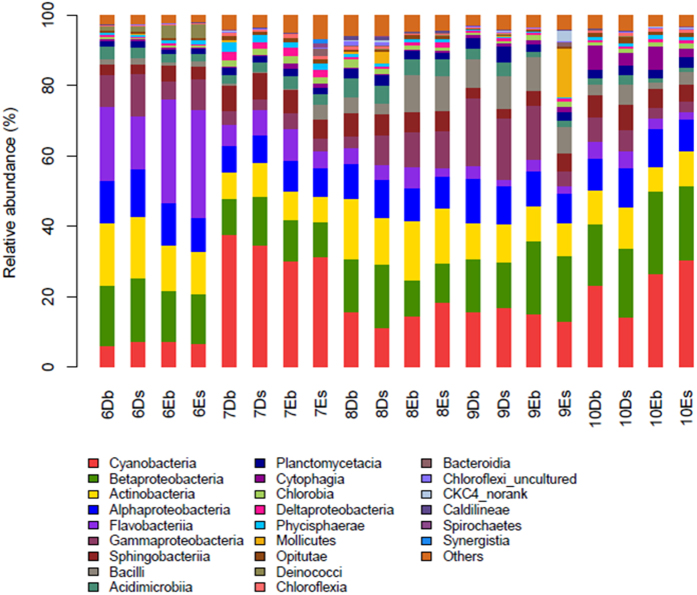
Relative abundance of bacterial community composition in twenty samples at the level of class over five months. Taxa represented occurred at >1% abundance in at least one sample.

To conduct a more detailed analysis of the composition of the communities in the water layers, all the reads had been assigned to a phylum into classes (Fig. 5). As observed, *Betaproteobacteria* were the dominant class among *Proteobacteria* in all water samples, accounting for 15.7%, followed by *Alphaproteobacteria*, *Gammaproteobacteria* and *Deltaproteobacteria*. Among these classes, *Gammaproteobacteria* were more abundant in D ponds (9.4% in surface layer, 8.5% in bottom layer) than in E ponds (5.9% in surface layer, 7.6% in bottom layer). However, the mean relative abundance of the five subdivisions within *Proteobacteria* in the two types of ponds was not significantly different over the five months (*p*  >  0.05). Interestingly, in D ponds, *Epsilonproteobacteria* were only detected in June and July, averaging at 0.006% in total effective sequences, whereas in E ponds, they were detected in June, July and August, averaging at 0.003%. In addition, *Deferribacteres* and *Thermotogae* were only detected in the surface water of E ponds in June.

**Figure 6:**
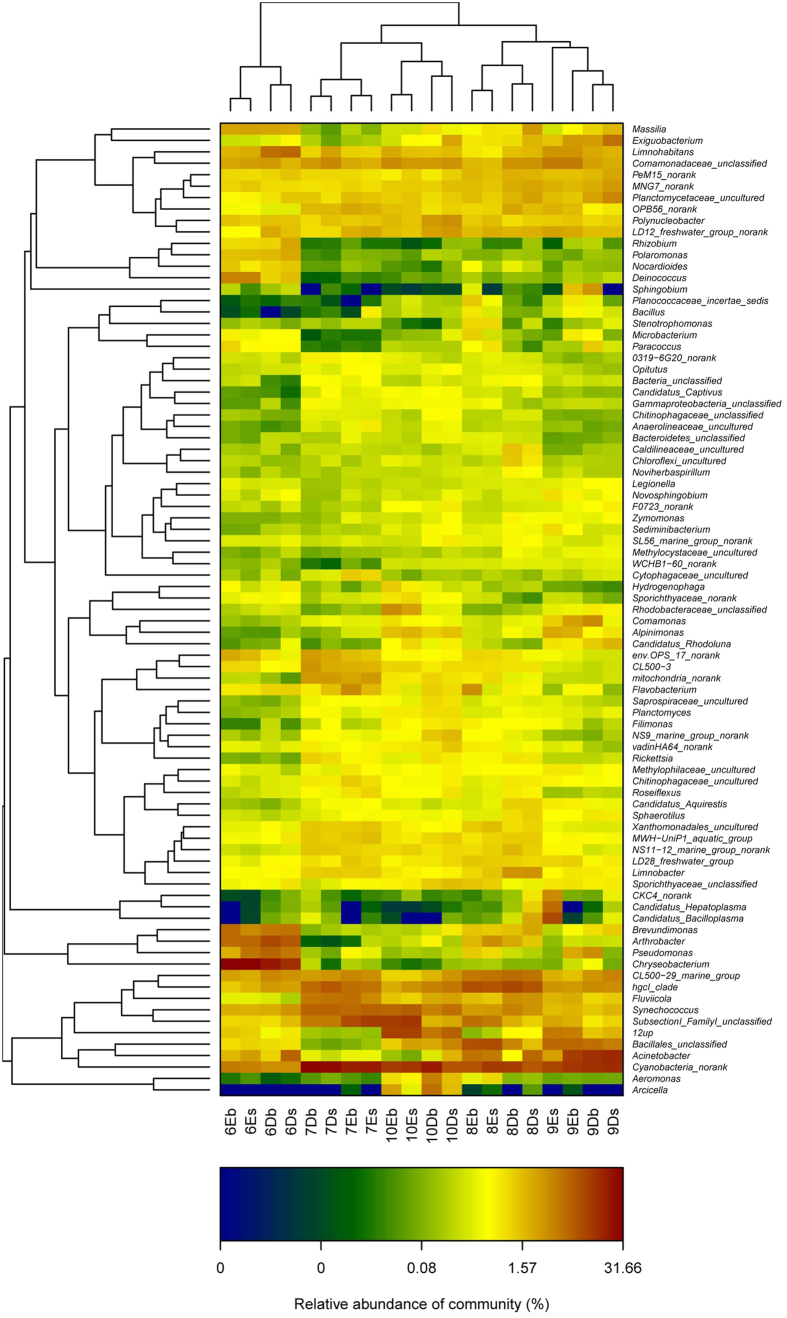
Heatmap showed the percentages of the abundant species in twenty samples at the level of genus over five months.

Hierarchically clustered heatmap showed the similarities and differences of these twenty bacterial communities at genus level (Fig. 6). Cluster analysis classified the samples into five clusters. Except for the samples from September, other samples from the same types of the pond were grouped together first, and then the samples in the same month from different types of the pond were clustered together.

At genus level, some interesting differences between the two types of ponds during the five months were observed. Some probiotics, such as *Comamonadaceae unclassified* and *Bacillales* unclassified were found to be significantly higher in D ponds than those in E ponds (*p* < 0.05) in July and August when the feed was changed to sudan grass feed. On the other hand, some potential pathogens such as *Acinetobacter* and *Aeromonas* were found to be significantly lower in D ponds than those in E ponds (*p* < 0.05) in August.

### Similarity analysis of the twenty water samples

**Figure 7:**
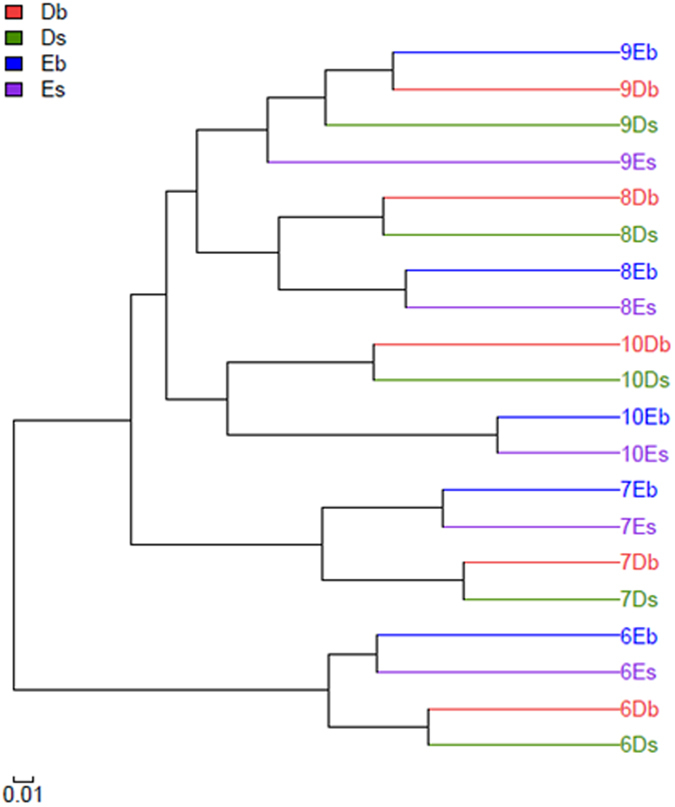
UniFrac clustering relationship estimated using OTU representative reads.

The weighted UniFrac clustering method was used to calculate the similarity or dissimilarity of the obtained sequences among different samples^25^. As shown in Fig. 7, based on abundances of orders, the bacterial communities in the twenty samples could be clustered into five groups, which included all four samples from one of the five months respectively: Group I (September), Group II (August), Group III (October), Group IV (July), Group V (June). Except for those from the samples in September, the communities from the same type of ponds were clustered together.

**Figure 8:**
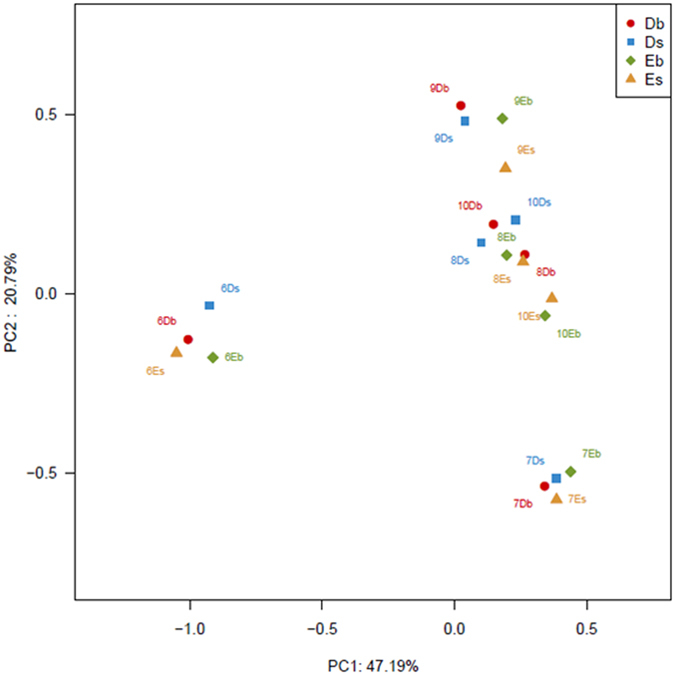
Principal coordinate analysis (PCoA) of twenty water samples by weighted UniFrac.

PCoA was used to estimate the similarities among different water samples using three different approaches: RDP Classifier taxa, OTUs and UniFrac, and PCoA (Fig. 8). The first principal coordinate of the weighted analysis accounted for 47.19% of the variation in the data. It clearly separated the samples in June from those in other four months. PC2 accounted for 20.79% of the variance in the bacterial communities.

### Microbial community composition in relation to environmental variables

**Figure 9:**
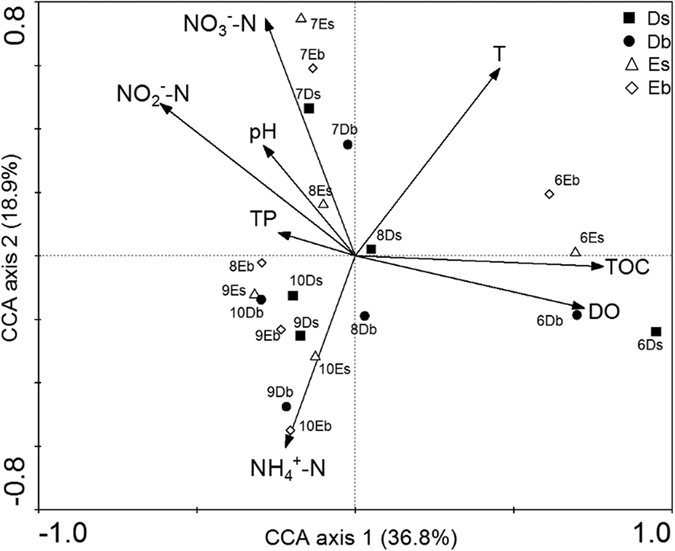
Canonical correspondence analysis (CCA) ordination diagram of bacterial communities associated with environmental variables of ammonia (NH_4_^+^-N), nitrite (NO_2_^−^-N), nitrate (NO_3_^−^-N), total phosphorus (TP), total organic carbon (TOC), temperature (T), pH, and dissolved oxygen (DO) (Eigenvalues: Axis 1: 0.385, Axis 2: 0.198, Axis 3: 0.168, Axis 4: 0.091). Environmental variables were indicated as arrows.

CCA was used to establish the relationships between the environmental factors and the bacterial community (Fig. 9). CCA plot was carried out using OUTs data together with environmental data (ammonia, nitrite, nitrate, total phosphorus, total organic carbon, temperature, pH, and dissolved oxygen). According to Monte Carlo permutation test (499 permutations), the significant relationships between environmental variables and canonical axes were analyzed by using Canoco program.

Based on the 5% level in a partial Monte Carlo permutation test, the bacterial community and structure were significantly linked (*p* <  0.05) to the water environment factors. As shown in Fig. 9, the water samples were clearly clustered according to sampling time rather than sampling water layer. CCA results explained 36.8% and 18.9% of the variation in the first two axes, respectively (Fig. 9). Dissolved oxygen, temperature and total organic carbon were the most important environmental factors to influence the water community composition, and were positively correlated with Axis 1 (*p* < 0.05) (Table 3). Axis 2 had a positive correlation with temperature, nitrite and nitrate (*p* < 0.05), but was negatively correlated with ammonia (*p* < 0.01) (Table 3), suggesting that Axis 2 had a gradient in temperature, nitrite, nitrate and ammonia. By contrast, other nutrient factors (pH and total phosphorus) had no significant correlation to bacterial community (*p* > 0.05).

## Discussion

In this study, we showed the vertical stratification of the 16S rRNA bacterial abundance in the water layers of two different types of aquaculture ponds. The bacterial 16S rRNA gene abundance was significantly higher in August than in other four months in both types of ponds. And the highest temperature was observed in August. These results indicate that temperature may play an important role in the seasonal dynamics of bacterial abundance. Similar phenomenon was observed in other aquaculture ponds, where total bacterial abundance varied with temperature^26^.

After changing the types of feeds, the bacterial abundance in E ponds was higher than that in D ponds (with sudan grass supplied in July and August) in September (*p* < 0.05) and October (*p* < 0.05), respectively. But there was no significant difference in bacterial abundance between D and E ponds in July and August. These results reveal that different types of feeds can influence the bacterial abundance, and possibly the effect of supplying sudan grass on the bacterial abundance was slightly delayed. The higher bacterial abundance in E ponds in September and October may be due to the trophic state of the ponds, as previous studies have demonstrated that the total number of bacterial cells observed in three different sediments may be influenced by the reservoir trophic state^27^.

The number of OTUs in each sample was estimated with the largest 16S rRNA libraries sequenced to date. However, a lot of “Unclassified” sequences were also detected in the samples, suggesting that there might be a high abundance of unknown microbial lineages in the aquaculture pond environment, which should be studied in detail in the future. At the 3% distance cutoff level, the OTUs ranged from 699 to 1153 in this study. The Shannon diversity values ranged from 3.97 to 5.31 and the highest was observed in 8Ds. These values are in accordance with those reported for some other aquaculture systems^28^,^29^. However, according to the study of the microbial diversity of seawater in the East China Sea, the Shannon diversity values were found in the range of 3.42 to 5.65^30^. Another study showed low diversity values, ranged from 2.4 to 3.4 when studying the microbial community of a warm monomictic tropical freshwater lake^31^. These results indicate that aquaculture system may harbor higher microbial species richness than other aquatic systems.

Seasonal pattern of bacterioplankton community composition has been reported for aquaculture systems^32^, river^33^, lake^34^, coastal water^35^ and the open ocean^36^. However, these previous studies were mostly based on fingerprinting methods, which only include the most abundant members of the community. The present study is the first attempt to apply Illumina MiSeq sequencing to study the seasonal dynamics, including both abundant and rare populations of the observed OTUs in two different types of aquaculture ponds. We showed the vertical stratification of the microbial communities in the pond water layers over five months, and two water layers corresponding to the surface and bottom water bodies were investigated. We hypothesized that there would be significant differences among the water samples from different months. It was shown that the studied microbial community was featured by strong temporal shifts and seasonal clustering. As demonstrated by the clustering, PCoA, and CCA analyses, the water samples from the same sampling month were fairly similar, while the samples from different months were different. This difference may be due to the difference in water conditions in different months, which determine the microbial community distribution. Previous studies have demonstrated that different temperatures and oxygen concentrations significantly alter the microbial community composition in freshwater sediment^37^,^38^. One potential driver of seasonal change in community composition is temperature^39^, and this can also be the case for pond water, as the growth of microbes in this study was at least seasonally affected by temperature. This result is consistent with the results of studies on phytoplankton communities, which showed that pelagic bacterial communities experienced climate-driven seasonal environmental changes^40^. The CCA result in our study also demonstrates that the temperature is positively correlated with the water community composition. Thus, temperature seems to be an important factor that affects microbial communities.

Many previous studies on the composition of microbial communities in the water have relied heavily on clone library analysis, which only sequences rather few16S rRNA gene fragments^11^,^41^,^42^. A previous study analyzed the bacterial diversity in the influent from a municipal waste water treatment plant using high-throughput sequencing method^43^. Their results showed that *Proteobacteria* were the most abundant phylum in the influent sample, which is consistent with our study. However, the clustering of some phyla in the present study is consistent with the findings of the study of the surface water samples in sewage treatment plants^44^, which revealed that the main phyla were *Proteobacteria*, *Actinobacteria*, and *Bacteroidetes* based on an examination of bacterial community structures by 454 sequencing, but the relative abundance differed from that in the present study. Their results showed that the dominant phyla were *Proteobacteria* (20.28–67.89%), *Bacteroidetes* (3.85–16.14%), *Acidobacteria* (19.78–53.59%), and *Cyanobacteria* (0.68 to 2.6%) in surface water samples, while our results revealed that *Proteobacteria* were the most abundant phylum in all twenty water samples and accounted for 24.28–48.22%, followed by *Cyanobacteria* (5.9–37.61%), *Bacteroidetes* (5.4–34.82%), and *Actinobacteria* (8.2–22.37%). Another study reported the bacterial community composition in the Red Sea based on 454 pyrosequencing, and revealed the vertical stratification of the microbial communities in the water layers above the Atlantis II and Discovery Deeps. Their study showed that the classified bacterial reads from the upper layers (20 and 50 m) were dominated by *Cyanobacteria*, whereas in the deeper layers (200 and 1500 m), the largest group was *Proteobacteria*^19^.

The microbial compositions at different depths seemed to be very similar in both types of ponds, and no significant differences of the four dominant phyla (*Proteobacteria*, *Cyanobacteria*, *Bacteroidetes*, and *Actinobacteria*) were observed between the surface and bottom layers. These results are similar to those of a study using 16S rRNA pyrosequencing in seawater^19^, which showed that the microbial composition in the surface (20 and 50 m) seems to be very similar. This phenomenon may be due to the similarity of water conditions in the surface and bottom water layers. Freshwater pond is a shallow water body, in which the nutrient concentrations showed no significant difference between the surface and bottom water layers as shown in Table 1. Similar results were also reported in intensive GIFT tilapia (*Oreochromisniloticus*) ponds^45^, which demonstrated that the microbial compositions of four different water layers were similar.

There were no significant differences in the four dominant phyla (*Proteobacteria*, *Cyanobacteria*, *Bacteroidetes* and *Actinobacteria*) between the two types of ponds over the five months. Similar phenomenon was also observed in two rivers, where two clone libraries of 16S rDNA constructed with summer samples from each river were not significantly different and contained typical freshwater bacterioplankton of *Betaproteobacteria*, *Bacteroidetes*, and *Actinobacteria*^33^. However, as demonstrated by the clustering and PCoA analyses, the communities from the same type of ponds were clustered together in this study, except for the samples in September. Besides, except for in the water samples in June, *Proteobacteria* were found to be the dominant phylum in the samples from other months, which is similar to the analytical results of bacterial communities in drinking water^46^. The classified bacterial reads of the samples from D ponds were dominated by *Proteobacteria*, which were decreased in E ponds in each depth (Fig. 5). *Proteobacteria* contain a very high level of bacterial metabolic diversity related to global carbon, nitrogen and sulfur cycling^47^. The effects of different types of feeds on the distributions of the proteobacterial subdivisions in the ponds were also studied. Here, *Betaproteobacteria* were the most abundant class in the *Proteobacteria* phylum. This is different from the results of a study using 454 pyrosequencing^19^, which showed that *Gammaproteobacteria* were the most abundant *Proteobacteria* in sea water. However, our finding is similar to the analytical results of bacterial communities in soil^48^ and activated sludge^18^, which demonstrated that *Betaproteobacteria* were the most abundant in *Proteobacteria*. The classified bacterial reads of the samples from D ponds were dominated by *Gammaproteobacteria*, which were decreased in samples from E ponds (Fig. 5). A previous study showed the shift of bacterial community structure to a relatively higher abundance of *Gammaproteobacteria* due to the addition of organic carbon substances^49^. In our study, the TOC concentration (Table 1) in D ponds was slightly higher than that in E ponds, which might result from the supply of sudan grass for D ponds in July and August. A decrease of *Actinobacteria* in the samples of E ponds was observed compared within the samples of D ponds. Here, both the nitrate and ammonium concentrations were higher in E ponds than in D ponds, which is supported by a study which demonstrated that both the nitrate and ammonium concentrations negatively affect the abundance of *Actinobacteria*^50^. *Bacteroidetes* were enriched in water samples of E ponds. This might be explained by the reason that the ammonia concentration was higher in E ponds than in D ponds, which is supported by a study showing that *Bacteroidetes* are positively correlated with ammonia concentration in a hot spring^51^.

*Comamonadaceae unclassifiedand* and *Bacillales*_unclassified were significantly enriched in D ponds compared with in E ponds when the feed was changed to sudan grass feed. *Comamonadaceae unclassified* was are likely to be nitrate-reducing bacteria^52^ and could decrease organic carbon in the ponds. Their higher abundance in D ponds might be related to the higher TOC concentration in D ponds resulted from the addition of available organic carbon for denitrification. Some species of *Bacillus* are used as probiotics, such as *Bacillus subtilis*, *Bacillus licheniformis*, in the aquaculture system. *Acinetobacter* and *Aeromonas*, many species of which are pathogenic bacteria in aquaculture ponds^53^,^54^, were significantly abundant in E ponds in August. This result indicates that sudan grass is effective to decrease *Acinetobacter* and *Aeromonas*. The reasons for the higher abundance of *Bacillus*, *Acinetobacter*, and *Aeromonas* in E ponds still need further study.

CCA in this study shows that DO, temperature and nutrients (TOC, nitrate, ammonium and nitrite) significantly influence the composition of bacterial communities in pond water. A previous study showed that DO, temperature, pH and nutrients (total nitrogen, total phosphorus) are the key factors influencing the bacterioplankton diversity of Lake Taihu^55^. Another study showed that ammonium, chemical oxygen demand and total nitrogen have effects on the composition of bacterial communities in *Litopeneaus vannamei* aquaculture water^56^. CCA plots revealed that samples collected in the same month were almost clustered together. This result suggests that the bacterial communities might demonstrate a seasonal pattern, which is consistent with the previous studies^57^,^58^.

In conclusion, the community structure differed between the ponds supplied with different types of feeds and the ponds supplied with sudan grass had better water conditions. The pathogenic bacteria genera *Acinetobacter* and *Aeromonas* were significantly decreased in the ponds supplied with sudan grass. In addition, the microbial communities in the ponds had a seasonal pattern in our study. These results suggest that the modulation of diets might influence the community structure and thus might effectively change water quality. Our findings could provide a promising direction for the healthy aquaculture of grass carp.

## Materials and Methods

All experiments were approved by and carried out in compliance with the guidelines of the Institutional Animal Care and Use Committees (IACUC) of Huazhong Agricultural University, Wuhan, China

### Fish culture and water sampling

Water samples were collected from an aquaculture farm (29°54′6.61″ N, 112°15′56.41″ E), Gong’an, Hubei, China. Five aquaculture ponds were selected for investigation. The ponds had a water surface of 2800 m^2^ (70 m × 40 m), with an average depth of 1.6 m and maximum depth of 1.8–2.0 m, where grass carp (*Ctenopharyngodon idellus*) were raised as the major species. At the time of sampling, the ponds had been used for 3 years with stable production under intensive cultural management. Five ponds differed mainly on the supply of fish feeds: three of them were provided with commercial feed from June to October 2014, and the other two were provided with commercial feed in June, September, October 2014, and sudan grass (*Sorghum sudanense*) in July and August 2014, respectively.

The sampling was conducted in 2014 on June 16^th^, July 16^th^, August 28^th^, September 21^st^, and October 14^th^. Samples were taken from the surface and bottom water column, which corresponded to 0.2 and 1.6 m of depth, respectively. Five samples (200 mL) were taken from five evenly distributed points within each pond then mixed for each depth. These water samples were collected, homogenized and sub-sampled for further analysis. One part of these water samples were used to measure the physicochemical factors immediately. The other part samples were placed in an incubator box with ice packs until further processing in the laboratory and these water samples were filtered through a 0.22-μm nucleopore filter (diameter: 47 mm) within 12 h after water collection, then kept at −80 °C until DNA extraction. The ponds supplied with sudan grass or commercial feed were designated as D ponds and E ponds, respectively. Thus, the water samples were designated as: Ds and Db (the surface and bottom water of D ponds, respectively), Es and Eb (the surface and bottom water of E ponds, respectively). The number before the sample name represents the sampling month (for example, 6Ds refers to the surface water sample of D ponds collected in June).

### Physicochemical analysis

Temperature (T), pH, and dissolved oxygen (DO) concentration were measured using an HQ 30d multi-parameter water quality analyzer (HACH, Loveland, CO). Total phosphorus (TP) was determined with ammonium molybdate spectrophotometric method. Total organic carbon (TOC) was determined on a Total Organic Carbon Analyzer (Elementar, Hanau, Germany). Ammonia (NH_4_^+^-N), nitrite (NO_2_^−^-N), and nitrate (NO_3_^−^-N) were determined by the methods described by Lu *et al*.^55^.

### Quantitative PCR (qPCR)

Total genomic DNA of each sample was extracted as previously described^59^. The concentration of the extracted DNA was quantified with a ND-2000 UV-vis spectrophotometer (USA). Quantitative PCR was carried out using a Qiagen Q thermo cycler (Qiagen, Hilden, Germany). The reaction mixture (20 μL) contained 10 μL SYBR Premix Ex Taq II, 0.2 μM of each primer and 1 μL template DNA (10 ng). The specific primers for the amplification of bacterial 16SrRNA genes were 515F (5′-GTGCCAGCMGCCGCGG-3′) and 907R (5′-CCGTCAATTCMTTTRAGTTT-3′) as previously described^60^. The qPCR reactions were performed as follows: 95 °C for 30 s; 35 cycles of 95 °C for 5 s, 55 °C for 30 s, followed by 72 °C for 1 min. The specificity of the qPCR amplification was determined by melting curve and gel electrophoresis.

The standard curve was constructed using plasmid DNA as the standard sample. The plasmid DNA was extracted from the positive colony, which was confirmed by that bacterial 16S rRNA genes were successfully ligated into the pMD18-T Vector (Takara, Dalian, China). The concentration of the plasmid DNA was determined with a ND-2000 UV-vis Spectrophotometer and bacterial 16S rRNA gene copy numbers of plasmid DNA were calculated using the detected concentration. The standard samples were produced by ten-fold serial dilution of plasmid DNA. The standard curve was observed to have a correlation coefficient (R^2^) of 0.995 and an efficiency of 93%.

### Illumina MiSeq sequencing

In this study, we collected samples for five consecutive months (from June to October) to conduct Illumina MiSeq sequencing. All PCR products of 16S rRNA genes were performed in triplicate using Illumina MiSeq Sequencer (Illumina) by Majorbio Bio-pharm Biotechnology Co. Ltd. (Shanghai, China) as described previously^61^. PCR products were pooled and purified using an Axy Prep™ DNA Gel Extraction Kit (Axygen). All the sequences used in this study are available from the NCBI Sequence Read Archive (SRA) under accession number SRA3016262.

### Bioinformatics and statistical analysis

Sequencing data were processed using the Quantitative Insights Into Microbial Ecology (QIIME) pipeline (http://qiime.sourceforge.net/)^62^. The sequences with the same barcode were assigned to the same sample, and then the barcode and primer sequences were removed. Denoised sequences with one mismatched base in the barcode, overlapped shorter than 10 bp, containing ambiguous characters, with more than two mismatched bases in the primers, or with a sequence length shorter than 50 bp were eliminated. The chimeric sequences were removed from aligned sequences using the UCHIME method^63^. And the valid reads obtained from Illumina MiSeq sequencing were normalized to 20000 for comparison of community diversity. The reads were then clustered into operational taxonomic units (OTUs, 97% similarity)^64^. The Greengenes data base was used to determine the taxonomic identity of each phylotype^65^. The diversity indices ACE and Chao1^66^ were estimated using Mothur^67^ to indicate the community richness. Simpson and Shannon indices were estimated to indicate the community diversity. Coverage, which represents the sequencing depth, was calculated using custom R scripts. The differences in overall bacterial community structure between each pair of water samples were detected using the UniFrac metric^25^. Heatmap was constructed with R software. PCoA (principal coordinate analysis) was used to characterize the changes of the community composition by using R software.

All data are presented as the means ± standard deviation (SD); n refers to the number of samples. Statistical analysis was performed using the SPSS 21.0 software package. The differences in the abundance of bacterial 16S rRNA genes and in the four dominant phyla among the samples from the two types of ponds were evaluated by one-way ANOVA. The differences between the relative abundance of genus from the two types of ponds were also evaluated by one-way ANOVA. CCA (Canonical correspondence analysis) plot and Monte Carlo Permutation test were used to analyze the relationship between the pond water community composition and the environmental factors using CANOCO 4.5 program.

## Additional Information

**How to cite this article**: Qin, Y. *et al*. Bacterial abundance and diversity in pond water supplied with different feeds. *Sci. Rep.* **6**, 35232; doi: 10.1038/srep35232 (2016).

## Supplementary Material

Supplementary Information

## Figures and Tables

**Table 1:** Environmental parameters of the surface and bottom water samples.

Sample name	Ds	Db	Es	Eb
T (°C)	24.4 ± 4.1	24.1 ± 4.3	24.3 ± 3.7	23.9 ± 4.5
pH	7.91 ± 0.25	7.92 ± 0.15	7.93 ± 0.14	7.87 ± 0.11
DO (mg/L)	3.89 ± 1.41	3.29 ± 1.53	3.18 ± 1.41	2.85 ± 1.47
NH_4_^+^-N (mg/L)	0.96 ± 0.30ab	0.83 ± 0.18ac	1.57 ± 0.90bd	1.51 ± 0.99bd
NO_3_^−^-N (mg/L)	0.25 ± 0.10	0.26 ± 0.98	0.42 ± 0.31	0.43 ± 0.32
NO_2_^−^-N (mg/L)	0.06 ± 0.05a	0.06 ± 0.05a	0.13 ± 0.08b	0.13 ± 0.09b
TP (mg/L)	0.12 ± 0.08	0.12 ± 0.07	0.12 ± 0.75	0.14 ± 0.11
TOC (mg/L)	15.6 ± 5.0	15.4 ± 4.5	15.5 ± 2.7	14.7 ± 2.3

All data are presented as means ± standard deviation (SD); n = 10, 10, 10, and 15 for Ds, Db, Es, and Eb, respectively. In the sample name, the capital letters D and E represent the ponds in which grass carp were fed with sudan grass and commercial feed, respectively; the lower case s and b represent the surface and bottom water layer, respectively. Lowercase letters (a–d) indicate significant differences between two groups (*p* < 0.05).

**Table 2:** Diversity indices from 20 water samples.

Sample name	Reads	3% Dissimilarity					
		OTU	ACE	Chao 1	Coverage (%)	Shannon	Simpson
6Ds	29378	720	926	925	99.3	4.51	0.03
6Db	25524	699	890	867	99.2	4.27	0.06
6Es	28007	729	974	993	99.2	3.97	0.09
6Eb	44641	820	1001	1006	99.5	4.11	0.09
7Ds	33939	941	1168	1155	99.2	5.08	0.02
7Db	27990	893	1076	1076	99.2	5.11	0.02
7Es	46441	1153	1308	1306	99.5	5.27	0.02
7Eb	42736	1118	1299	1305	99.4	5.21	0.02
8Ds	25552	933	1141	1134	99.1	5.31	0.01
8Db	31916	1016	1231	1230	99.2	5.28	0.01
8Es	42707	1040	1251	1237	99.4	5.01	0.02
8Eb	34433	1003	1230	1229	99.3	5.17	0.02
9Ds	27911	884	1127	1104	99.1	4.75	0.03
9Db	33261	936	1196	1237	99.2	4.78	0.02
9Es	40029	956	1179	1228	99.4	4.83	0.02
9Eb	35568	937	1144	1141	99.3	4.89	0.02
10Ds	27350	903	1109	1110	99.1	5.24	0.01
10Db	23041	840	1071	1073	98.9	4.95	0.02
10Es	45949	1037	1241	1218	99.4	4.78	0.03
10Eb	47566	999	1156	1126	99.6	4.8	0.03

The numbers of reads after quality control and noise clearance are shown. The number before the sample name represent the sampling month. In sample name, the capital letters D and E represent the ponds in which grass carp were fed with sudan grass and commercial feed, respectively; the lower case s and b represent the surface and bottom water layer, respectively.

**Table 3:** Weighted correlation matrix of environment factors with CCA axis.

Environmental factors	Axis 1	Axis 2
pH	−0.28	0.33
DO	0.69**	−0.15
T	0.43*	0.55**
NH_4_^+^-N	−0.21	−0.57**
NO_2_^−^-N	−0.59**	0.45*
NO_3_^−^-N	−0.27	0.70**
TP	−0.23	0.07
TOC	0.74**	−0.032
