# Supplementary material for: Bacterial abundance and diversity in pond water supplied with different feeds
Source: Sci Rep. 2016 Oct 19;6:35232. doi: 10.1038/srep35232 (PMC5069485; doi:10.1038/srep35232)
Supplement: Supplementary Information [file srep35232-s1.pdf]

Bacterial abundance and diversity in pond water supplied with different feeds

Ya Qin[1], Ming Deng[1], QuanshengLiu[1,2], Chongwei Wu[1], YingjieJi[1] , Jie Hou[1,2,3] & Xugang He[1,2,3]

[1]Fisheries College, Huazhong Agricultural University, Wuhan 430070, China. [2]Freshwater Aquaculture Collaborative Innovation Center of Hubei Province, Wuhan 430070, China. [3]Hubei Provincial Engineering Laboratory for Pond Aquaculture, Wuhan 430070, China. Correspondence and requests for materials should be addressed to X.H. (email: xgh@mail.hzau.edu.cn) or J.H. (email: shengwuhj@mail.hzau.edu.cn)

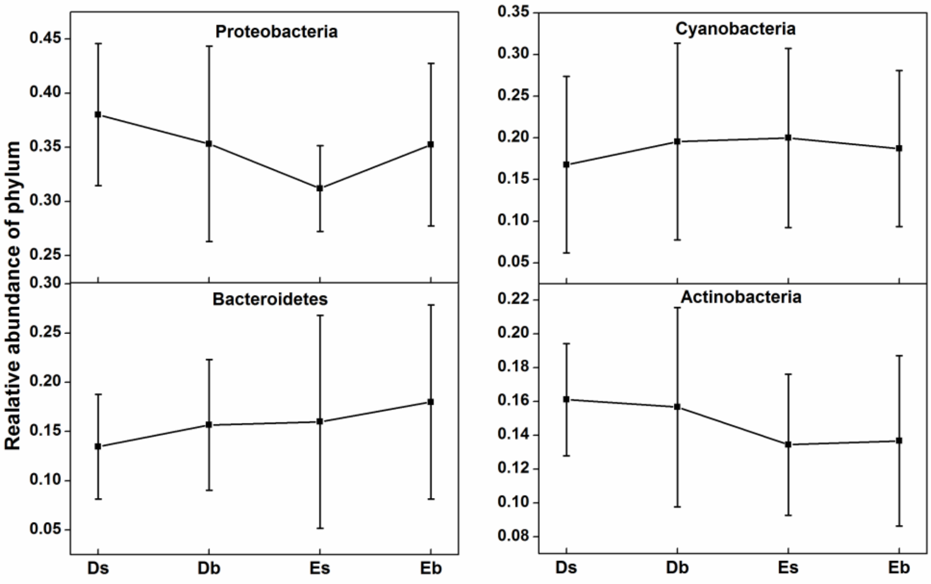

Supplementary Fig. S1 Mean relative abundance of the four dominant OTUs (*Proteobacteria*, *Cyanobacteria*, *Bacteroidetes* and *Actinobacteria*, respectively) for the five months at each sampling site. Error bars indicate standard deviations of the relative abundance for the five months (The capital letters D and E represent the ponds in which grass carp were fed with sudan grass and commercial feed, respectively. The lower case s and b represent the surface and bottom water layer, respectively).
